# Towards a therapy for mitochondrial disease: an update

**DOI:** 10.1042/BST20180134

**Published:** 2018-10-09

**Authors:** Caterina Garone, Carlo Viscomi

**Affiliations:** MRC-Mitochondrial Biology Unit, Cambridge CB2 0XY, U.K.

**Keywords:** bypass therapy, gene therapy, mitochondrial biogenesis, mitochondrial dysfunction, rapamycin

## Abstract

Preclinical work aimed at developing new therapies for mitochondrial diseases has recently given new hopes and opened unexpected perspectives for the patients affected by these pathologies. In contrast, only minor progresses have been achieved so far in the translation into the clinics. Many challenges are still ahead, including the need for a better characterization of the pharmacological effects of the different approaches and the design of appropriate clinical trials with robust outcome measures for this extremely heterogeneous, rare, and complex group of disorders. In this review, we will discuss the most important achievements and the major challenges in this very dynamic research field.

## Introduction

The extreme genetic, biochemical, and clinical complexity of primary mitochondrial diseases challenges both clinical and research activity in the field. Mutations in any of the mitochondrial genes encoding the 13 core subunits of the respiratory chain complexes and the 22 mitochondrial tRNAs and two rRNAs, as well as in any of the nuclear genes encoding the rest of the ∼1500 proteins constituting the mitochondrial proteome, may potentially lead to a mitochondrial dysfunction and disease. These orders can be transmitted with any kind of inheritance (recessive, dominant, X-linked, and mitochondrial) and can be characterized by multisystemic or organ-specific dysfunction that can arise at any time in life. This tremendous heterogeneity, together with a limited information on the natural history of the disease and a general lack of appropriate models, prevented, so far, the development of effective therapies.

In mtDNA-related disorders, the heterogeneity is partially explained by the degree of heteroplasmy, i.e. the relative load of mutant vs. wild-type mtDNA. For instance, the same mutation m.8993T>G in the ATPase 6 gene leads either to a childhood-onset of neuropathy and retinitis pigmentosa when the mutation load is ∼70% or to a fatal early-onset maternally inherited Leigh disease (MILS) when the mutation load exceeds 90% [1]. A high degree of clinical and biochemical heterogeneity is also observed in the presence of homoplasmic mutations, such in LHON (Leber's hereditary optic neuropathy) disease. LHON is one of the most frequent mitochondrial diseases and is due to homoplasmic mutations in mtDNA leading to blindness. The non-synonymous mutations at positions 11 778 in ND4, 3460 in ND1, and 14 484 in ND6 account for 90% of the patients [2]. The disease is characterized by incomplete penetrance, male prevalence, and spontaneous partial recovery of visual acuity. In addition, environmental factors and polymorphisms in mtDNA haplogroups J1c and J2c are also associated with increased penetrance of the disease [3]. Similarly, mitochondrial disorders due to defects in nuclear-encoded proteins can present as a disease spectrum in the presence of the same pathogenetic gene variant. In a recent study, the natural history of a large cohort of patients with confirmed diagnosis of TK2 deficiency was analysed. Three phenotypes with divergent survival were recognized (infantile, childhood, and late onset myopathy) based on the age at onset, rate of weakness, and post-onset survival and independently by the genotype. Indeed, the most common defect, p.Thr108Met, was responsible of the three different phenotypes in different families [4]. These unique features make extremely difficult the identification of outcome measures and clinical end-points in clinical trials.

At present, the therapies for mitochondrial disorders are limited to the treatment of the complications and supportive care with cocktails of vitamins (e.g. thiamine, riboflavin, folinic acid, and others), CoQ, aminoacids (arginine), lipoic acid, and other components. However, the efficacy of these supplements lacks solid preclinical or clinical evidence [5,6].

**Figure 1.**
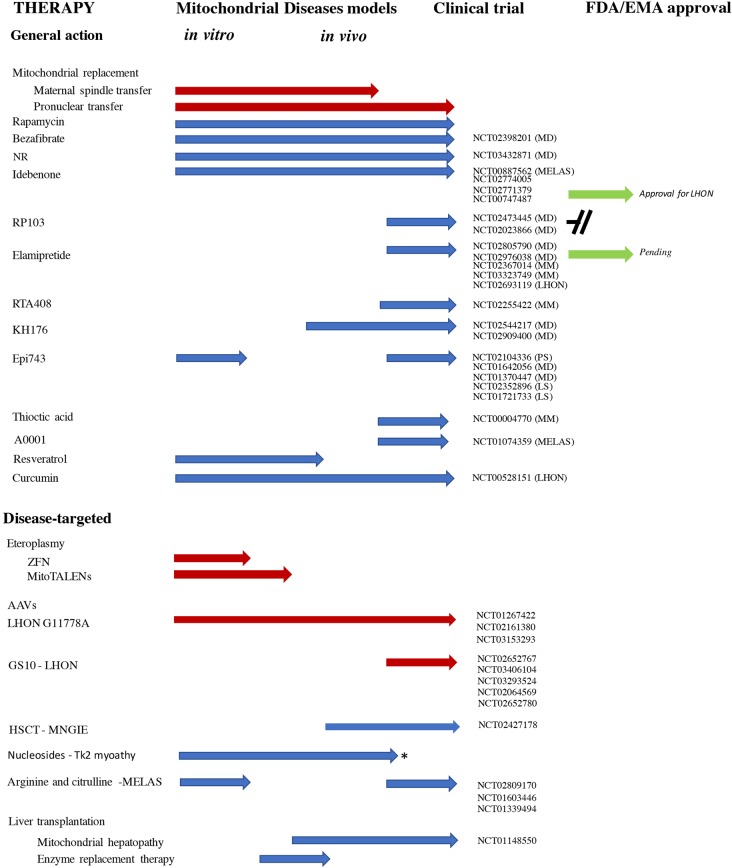
Development of new therapies for mitochondrial disorders. Preclinical (*in vivo* and *in vitro*), clinical and drug approval stages are represented in the figure. Note the lack of preclinical data in mitochondrial disease models for some new or re-purpose therapies. Treatments are also divided into ‘general action' or ‘disease target'. When clinical trials have been initiated, the clinicaltrial.gov code number is provided with the potential therapeutic indication. MM, mitochondrial myopathy; MD, mitochondrial disorder; LHON, Leber hereditary optic neuropathy; Tk2, thymidine kinase 2 deficiency; MELAS, mitochondrial myopathy, encephalopathy, lactic acidosis, stroke-like episodes; PS, Pearson syndrome; Red arrow, gene therapy; Green arrow, drug approval process; Blue arrow, drug compound; *= GMP product development.

Since the first edition of this review, many new publications introduced new concepts, opening new possibilities for treatment. In parallel, the number of clinical trials is steadily increasing. These developments are the subject of this review and are summarized in Figure 1.

## mTORC1: a new target for mitochondrial disorders

### Rationale

**Figure 2.**
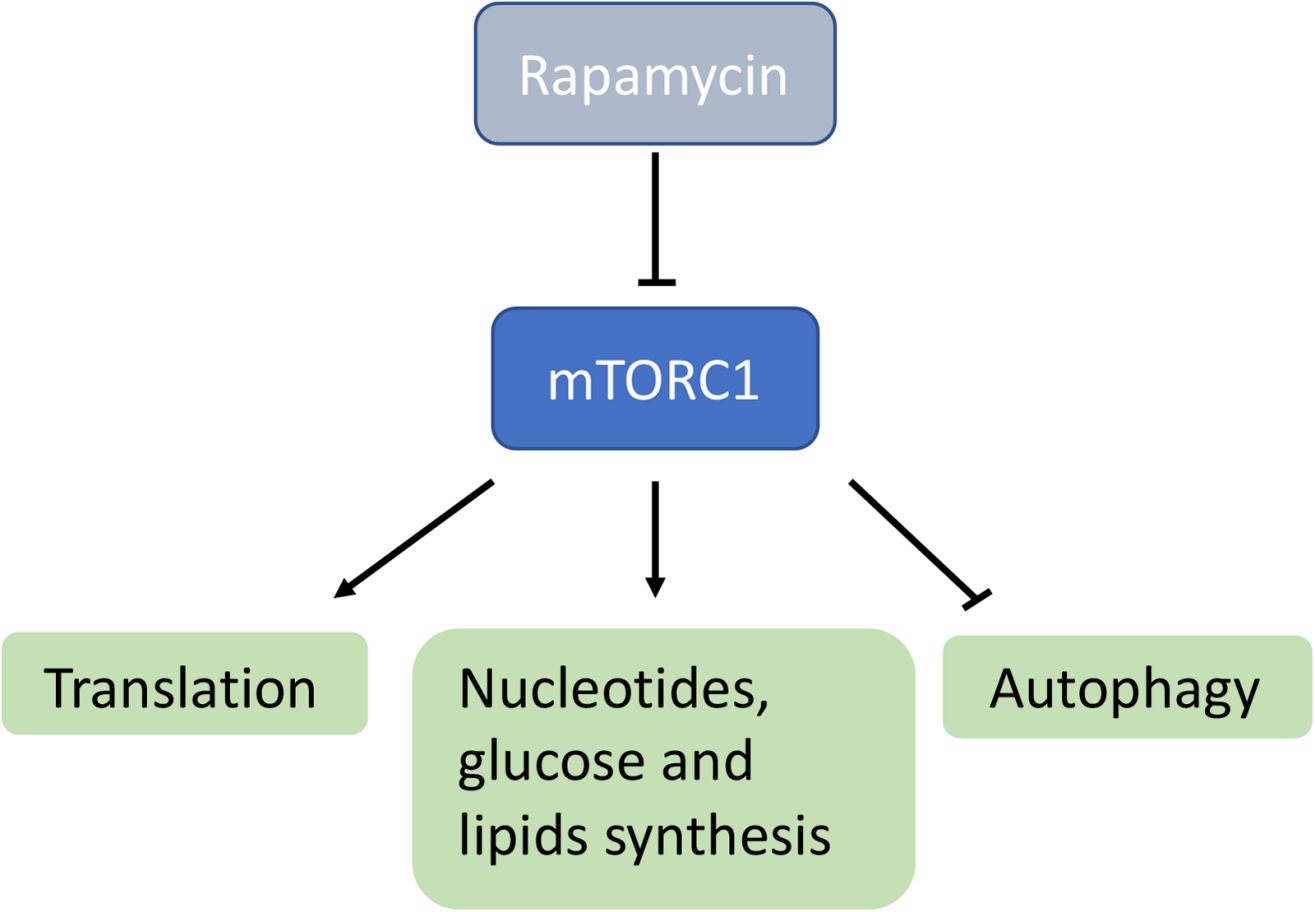
Scheme of the mTORC1-dependent metabolic pathways.

mTORC1 is a cytosolic Ser/Thr kinase belonging to the phosphatidylinositol kinase-related protein kinases family with central roles in several cellular processes, including protein translation, immune response, nucleotide and lipid synthesis, glucose metabolism, autophagy, and lysosomal biogenesis (Figure 2) [7]. The idea of using rapamycin, a widely used mTORC1 inhibitor, to treat mitochondrial diseases stemmed from the observation that inhibiting cytosolic translation significantly prolonged (approximately from 15 to 27 days) the replicative lifespan (i.e. the number of daughter cells a yeast cell can generate before exiting the cell cycle) of mitochondrial mutants in *Saccharomyces cerevisiae* [8].

### Results

Rapamycin (8 mg/kg i.p. starting 10 days after birth) markedly ameliorated the clinical phenotype and prolonged the median lifespan (from 50 to 110 days) of a knockout mouse for *Ndufs4* (*Ndufs4* KO), encoding the 18 kDa subunit of respiratory complex I, characterized by rapidly progressive encephalopathy resembling LS [9]. Rapamycin induced an extensive metabolic remodelling, including a shift from glycolysis to amino acid catabolism, the meaning of which remains, however, unclear.

Several subsequent studies aimed at better characterizing the mechanism of action. In one of these, post-onset *Ndufs4* KO mice were orally administered with rapamycin, with the dose kept the same as previously used in i.p., i.e. 8 mg/kg. This treatment delayed the development of the encephalopathy, in spite of blood steady-state levels post-treatment lower than those detected after systemic injection (32 vs. 45 ng/ml) [10].

In another study, rapamycin robustly increased by ∼34% the lifespan and rescued a fat storage defect in a *ND2*-deficient *Drosophila* model of LS, without affecting behavioural phenotypes and in an autophagy-independent manner [11]. However, the effect of rapamycin treatment on complex I activity was not investigated.

Rapamycin-treated iPSCs-derived neurons from a patient with MILS, carrying a mutation in the MT-ATP6 gene associated with reduced ATP synthesis, showed increased resistance to glutamate toxicity, possibly via inhibition of translation, thus preserving cellular ATP levels [12]. Similarly, inhibition of translation by mTORC1 inhibitors rapamycin and probucol, and by cycloheximide partially rescued the clinical and/or biochemical phenotypes of different models of mitochondrial dysfunction [13]. These included: (i) the CoQ-deficient mouse *B6.Pdss2^kd/kd^*, characterized by complexes I–III and II–III deficiencies, (ii) the gas-1(fc21) nematodes, carrying a homozygous mutation in the complex I *NDUFS2* subunit homologue, and (iii) rotenone-treated human cell lines mimicking complex I deficiency. Reduced ATP consumption and proteotoxic stress and activation of autophagy were shown to contribute to the overall effect [13].

In the Deletor mouse, carrying a dominant mutation in the mitochondrial helicase Twinkle, rapamycin down-regulated several components of the mtISR, a complex tissue-specific pathway involving transcriptional and metabolic adaptations, including the induction of the mitokines FGF21 and GDF15, one-carbon metabolism and mitochondrial unfolded protein response [14,15].

Low-dose rapamycin (0.8 mg/kg) administered to the mothers in drinking water before birth and increased to 4 mg/kg after birth, significantly prolonged by 60% the lifespan of *Tk2* knockin mouse model (*Tk2^H126N^*), defective for the pyrimidine-specific mitochondrial thymidine kinase [16]. This effect was due neither to the correction of mtDNA depletion nor to metabolic effects in the brain. In contrast, rapamycin induced significant changes in the liver in amino acid, carbohydrate, fatty acid, cofactor, and nucleic acid metabolism, without affecting key pathways of mitochondrial function, such as glucose, lactate, pyruvate, and β-hydroxybutyrate [16].

Notably, rapamycin was beneficial in all the models of mitochondrial disease tested, independently of the genetic lesion. Although the mechanism ultimately mediating rapamycin-dependent phenotypic amelioration of OxPhos-defective models is still highly debated, we can speculate that activation and/or inhibition of several pathways can contribute to the overall effect, including the inhibition of translation, which results in reduced energetic demand, and stress responses as well as the activation of autophagy.

### Outlook

Rapamycin is an approved drug used as immunosuppressant, and repurposing would be relatively straightforward, although appropriate investigations on mitochondrial patients would be required. In addition, several rapamycin analogues (rapalogues) with different mechanisms of action on mTORC1 are being developed [17], but have not been tested on mitochondrial disease models. Given the broad effects of mTOR inhibition, including immunosuppressive action, side effects are a major concern for the use of these compounds. It should be noted, however, that inhibition of mTOR with a rapalogue improved immune response to influenza vaccine, suggesting that compounds with a better safety profile may become available.

## Ketogenic diet: selecting against high mutational load

### Rationale

Ketogenic treatment (i.e. low glucose, high ketone bodies) was shown to shift heteroplasmy in cybrid cell lines carrying deleted mtDNA. Although the mechanism of this shift is unclear, a selective stimulation wild-type vs. mutant mtDNA replication has been proposed [18].

### Results

The hypothesis that ketogenic diet (KD) could induce a shift in heteroplasmy levels was tested *in vivo* in the Deletor mouse [19]. KD treatment decreased the amount of cytochrome c oxidase-negative muscle fibres, prevented the formation of the mitochondrial ultrastructural abnormalities in the muscle and reversed some of the metabolic changes observed in the mutant mice, possibly by stimulation of mitochondrial biogenesis. More recently, the results of KD treatment with a modified Atkins diet, a type of KD, in patients with mitochondrial myopathy and progressive external ophthalmoplegia with single or multiple deletions were reported [20]. All five patients showed signs of rhabdomyolysis within 2 weeks from the start of the treatment, confirmed by the damage to muscle fibres observed using electron microscopy. These results determined the interruption of the trial. Surprisingly, a 2-year follow-up revealed a relevant increase in muscle strength, suggesting a damage-induced stimulation of muscle repair by satellite cells, which do not carry deleted mtDNA molecules, following acute damage by Atkins diet.

In another study, reduced glucose intake to levels similar to that of KD into neuronal-like cybrids of MELAS was shown to reduce the accumulation of cI-subassemblies and to increase respiration along with mitochondrial content [21], although it is unknown how low glucose medium can induce mitochondrial biogenesis.

### Outlook

The study by Ahola et al. gave unexpected results, which suggest that KD-induced damage may trigger the activation of satellite cells, which are mtDNA deletion free, and can thus repair the skeletal muscle. More work is needed to investigate whether a modified regimen, based for instance on cycles of ketogenic and normal diet, may have a more robust effect [22].

## Hypoxia: the unexpected therapeutic option

### Rationale

**Figure 3.**
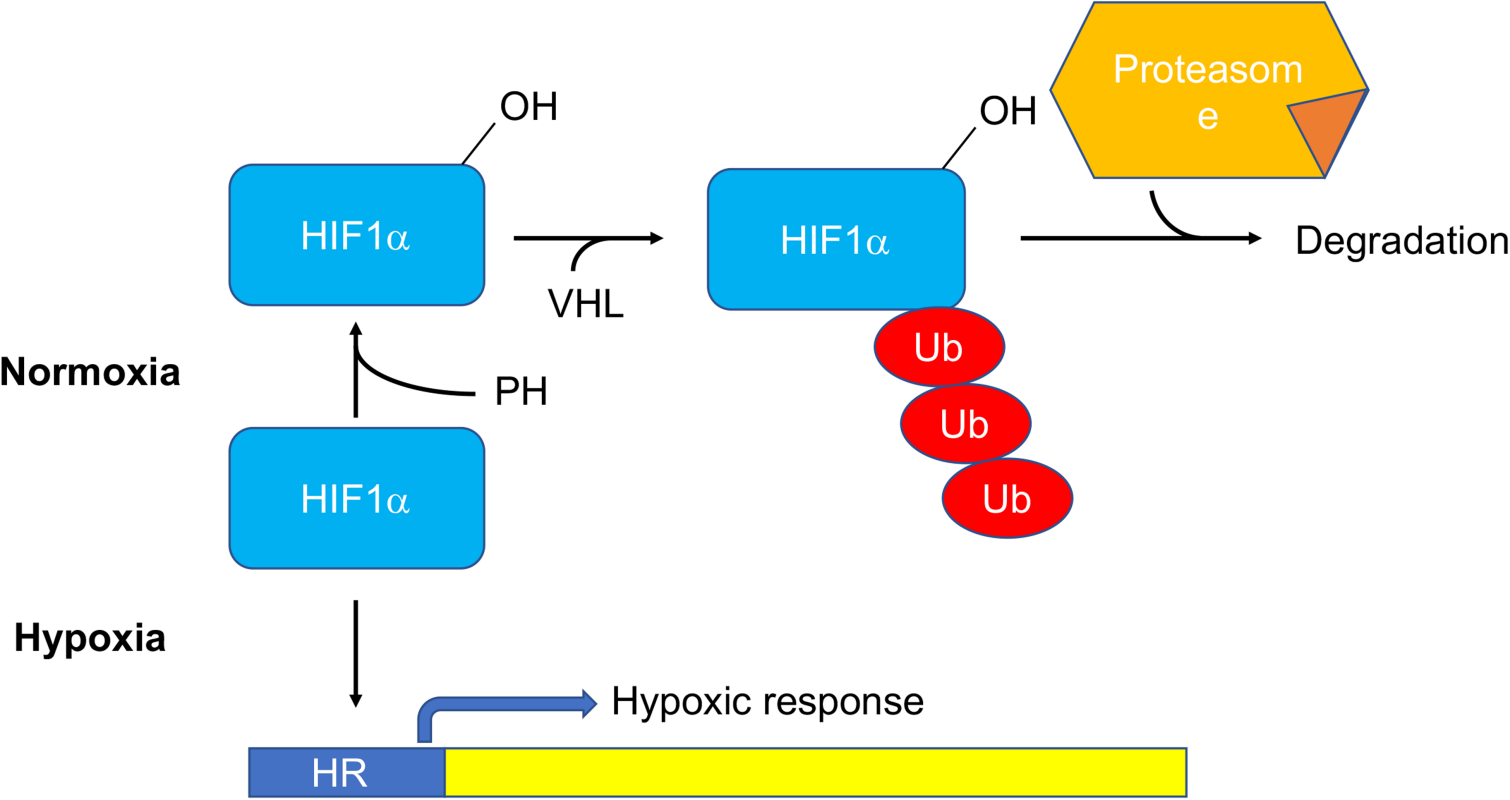
Regulation of the hypoxic response by stabilization of HIF1α. In normoxic conditions, HIF1α is hydroxylated by PH, ubiquitinated by the VHL, and thus targeted to the proteasome for degradation. During hypoxia, PH-dependent hydroxylation is blocked and HIF1α stabilized, thus activating the hypoxic transcriptional response. PH, prolyl hydroxylase; VHL, von Hippel–Lindau factor; Ub, ubiquitin.

In 2016, Jain et al. [23] identified the von Hippel–Lindau (VHL) factor, a major player in hypoxic response, as the most effective suppressor of antimycin-induced mitochondrial dysfunction in a Cas9-mediated screening. VHL is as an ubiquitin ligase that recognizes and targets for degradation the hydroxylated forms of the hypoxia-induced transcription factors (HIFs) [24]. During hypoxia, HIFs are stabilized because the hydroxylation reaction operated by the prolyl-hydroxylases stops (Figure 3).

### Results

Chronic normobaric hypoxic conditions (11% O_2_), started at 30 days, prevented the development of the disease in *Ndufs4* KO mice, drastically ameliorating the growth curve, the body temperature and the performance in neurological tests, which were deeply impaired in *Ndufs4* KO littermates exposed to normoxic conditions. Median lifespan was remarkably increased from 58 to 270 days. The neuropathological lesions of *Ndufs4* KO mice in olfactory bulbs, cerebellum, and brainstem were prevented by hypoxic conditions. In contrast, hyperoxia (55% O_2_) had the opposite effect and worsened all the parameters analysed [23]. Importantly, more moderate hypoxic conditions (17% O_2_) or alternate hypoxia/normoxia failed to improve the phenotype in *Ndufs4* KO mice [23]. In addition, return to normoxia rapidly reversed the beneficial effects, while hypoxic conditions established after the onset of the encephalopathic symptoms were able to reverse the brain lesions [25].

### Outlook

The fascinating results on the *Ndufs4* KO mouse await confirmation in additional models of mitochondrial disease. In addition, the mechanistic details are still unclear since hypoxia is likely to act at different levels, including: (i) metabolism, through the HIF-dependent transcriptional programme, which activates glycolysis diverting electrons from the defective respiratory chain decreasing ROS production; (ii) oxygen supply to the cells, blunting ROS production and signalling; (iii) organ physiology (e.g. O_2_ delivery and CO_2_ clearance, endocrine functions, immune response) [26]. Clarifying which pathways mediate the effect will open the possibility of pharmacologically activating them.

## Reducing ROS: between hopes and risks

### Rationale

Antioxidants are routinely used in the therapy of mitochondrial diseases under the assumption that increased ROS-related damage is a component of the pathogenesis. However, little evidence from preclinical studies is available on their efficacy. Only recently, some experimental work started to address this issue using cellular and *in vivo* models of mitochondrial dysfunction.

### Results

KH176, a derivative of Trolox acting both as antioxidant and redox modulator acting on the thioredoxin/peroxiredoxin system [27], was shown to improve rotarod performance and gait abnormalities with retention of the brain structure in the *Ndufs4* KO mouse [28]. KH176 is currently under clinical trial (Figure 1), and the first results covering safety, tolerability, and pharmacokinetics were recently published. These show that this drug was well tolerated up to single doses of 800 mg and multiple doses of 400 mg b.i.d. and had a pharmacokinetic profile supportive for a twice daily dosing. Only at high doses, KH176 causes clinically relevant cardiotoxicity [29].

NAC (*N*-acetyl cysteine) and vitamin E fully rescued, while CoQ, lipoic acid, orotic acid, and vitamin C partially prolonged the lifespan of gas-1(fc21) worms [30], possibly via reducing global oxidative stress since no correction of mitochondrial function was observed [30]. An additional study showed that in zebrafish the antioxidant probucol rescued embryo developmental delay induced by complex I inhibition with rotenone and complex V inhibition by oligomycin but not complex IV by azide [31]. The reasons for this difference are unclear.

NAC and ascorbate decreased ROS production in fibroblasts from a patient with complex IV-deficiency, mtDNA instability, and Fanconi anaemia due to mutations in COX4l1, encoding the common isoform of COX subunit 4 (COX 4-1) [32].

In spite of these not always coherent data, antioxidants remain central in clinical practice, and several antioxidants-based clinical trials, such as RTA408, idebenone, thioctic (lipoic) acid, EPI-743, KH176, are ongoing on different mitochondrial diseases (Figure 1). However, with the exception of KH176, none of these studies was supported by rigorous preclinical studies, and for only those based on idebenone the results have yet been released [33]. Notably, idebenone has been approved by EMA and an international consensus statement established the indication for the treatment in patients with acute, subacute or dynamic clinical course while did not recommended the treatment for chronic patients [34].

### Outlook

Antioxidants are probably the most widely used drugs in the therapy of mitochondrial diseases. However, there is accumulating evidence that antioxidants may also be detrimental in some conditions because they may interfere with ROS signalling. For instance, expression of alternative oxidase, an enzyme transferring electrons from CoQ directly to molecular oxygen in plants and lower eukaryotes, as well as the administration of NAC, significantly reduced lifespan of a muscle-specific Cox15 mouse model of severe mitochondrial myopathy by disrupting ROS-dependent mitochondrial biogenesis and satellite cell recruitment [35]. Future investigations are warranted to titrate the beneficial and toxic effect of antioxidants in different conditions and to better understand ROS signalling.

## Bypassing mtDNA replication enzymatic defects

### Rationale

**Figure 4.**
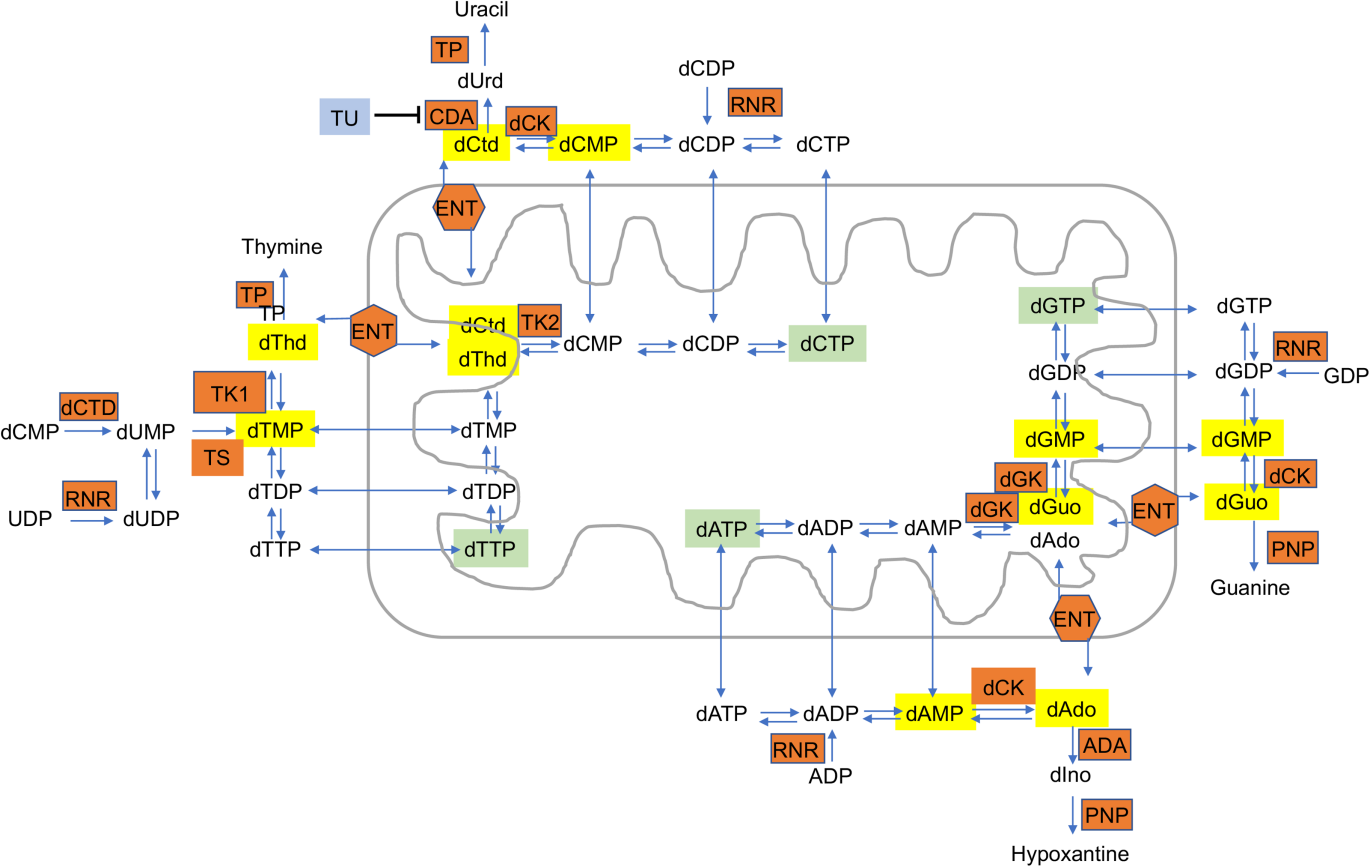
Representation of the main dNTP metabolic pathways. Catabolic enzymes are marked in orange boxes. ADA, adenosine deaminase; CDA, cytidine deaminase; dAdo, deoxyadenosine; dCK, deoxycytidine kinase; dCtd, deoxycytidine; dCTD, deoxycytidylate deaminase; dGK, deoxyguanosine kinase; dGuo, deoxyguanosine; dIno, deoxyinosine; dThd, thymidine; dUrd, deoxyuridine; ENT1, equilibrative nucleoside transporter 1; PNP, purine nucleoside phosphorylase; RNR, ribonucleotide reductase; THU, tetrahydrouridine; TK1, thymidine kinase 1; TK2, thymidine kinase 2; TP, thymidine phosphorylase; TS, thymidylate synthase. The dNTP precursors used in experimental setups to correct mtDNA instability are marked in yellow. The mitochondrial dNTP pools are marked in green. The enzymes are marked in orange.

Syndromes characterized by mtDNA instability are usually due to defects in enzymes directly involved either in mtDNA synthesis or in deoxynucleotide triphosphate (dNTP) metabolism. An imbalance of the nucleotide pools may trigger mtDNA instability. The supplementation of the missing or insufficient dNTP may thus be beneficial bypassing the block and restoring the dNTP pools (Figure 4). dNTPs are the building blocks for mtDNA synthesis and repair. They are supplied either by *de novo* synthesis and import from the cytosolic pool or by the mitochondrial deoxyribonucleoside salvage pathway (reviewed in [36]).

Defects in the enzymes responsible for dNTP pool maintenance perturbed mitochondrial DNA replication causing reduced copy number of mtDNA, multiple deletions, or point mutations of mtDNA and consequently affecting OXPHOS activities. Clinically, they present as a spectrum of disorders that ranges from severe infantile hepatocerebral, encephalopathy, or myopathy disorders to childhood-onset myopathy or adult-onset PEO.

### Results

Supplementation of deoxyribonucleotides and deoxyribonucleosides has been exploited in *in vitro* and *in vivo* models of mitochondrial dNTP pool unbalance. Molecular bypass therapy with deoxypyrimidine monophosphates (dCMP and dTMP) or substrate enhancement therapy with deoxypyrimidine nucleosides (dC and dT) orally administered to the *Tk2^H126N^* mice led to increased mtDNA levels and mitochondrial respiratory chain enzyme activities and prolongation of the lifespan of the homozygous mutant mice in a dose-dependent manner [37,38]. Deoxyguanosine alone corrected mtDNA depletion in *DGUOK*-deficient human fibroblasts, lacking the guanosine kinase phosphorylating purines in the salvage pathway [39]. Administration of pyrimidine and purine nucleosides has shown the ability to correct ethidium bromide-induced mtDNA depletion in human fibroblasts deficient for *RRM2B*, while their monophosphate form failed to correct depletion in *RRM2B*-deficient human myoblast [40,41]. Depletion of mtDNA has been corrected *in vivo* in a *Tymp/Upp1* double knockout mouse model of MNGIE disease by administrating deoxycytidine or tetrahydrouridine. In the same study, the addition of deoxycytidine and tetrahydrouridine to a cell model of MNGIE disease was also able to prevent mtDNA depletion [39].

### Outlook

Treatments with nucleotide and nucleoside monophosphates have been already translated for human compassionate use under FDA emergency IND and local ethic committee approval in 16 patients with TK2 deficiency. The preliminary data are expected soon.

## Increasing mitochondrial biogenesis

### Rationale

Since bioenergetic defects and reduced ATP synthesis play major roles in the pathogenesis of mitochondrial disease, increasing mitochondrial mass and/or activity can, in principle, be beneficial. The stimulation of mitochondrial biogenesis is regarded as one of the most promising approaches for mitochondrial disease [42,43].

### Results

Positive results have been reported on several mouse models using the AMPK agonist AICAR [44], and compounds increasing NAD^+^ concentration, such as the NAD^+^ precursor NR [45,46] and the inhibitors of NAD^+^ consuming enzymes (e.g. PARP1) [45]. In contrast, no beneficial effect was observed in the *BCS1L* KO mouse, a model of complex III deficiency with a predominant liver disease [47]. However, it should be noted that the mitochondriogenic effect of PGC1α stimulation has been mainly described in brown adipose tissue and skeletal muscle, while in the liver it has been associated with the stimulation of gluconeogenesis [48].

Interestingly, increased mitochondrial content has been shown to be protective in non-manifesting carriers of the LHON mutations, opening the hypothesis of a role in the incomplete penetrance of the disease [49]. Another study suggested that oestrogens have a protective role against LHON, possibly by increasing mitochondrial biogenesis [50]. In addition, the same study showed that the stimulation of the mitochondrially localized oestrogen receptor β by using the phyto-oestrogen 17β-oestradiol improved cell viability by reducing apoptosis, inducing mitochondrial biogenesis and strongly reducing the levels of ROS. These data open the possibility of using phyto-oestradiol topically in LHON.

### Outlook

Pharmacological compounds stimulating the mitochondrial biogenesis are currently under clinical trial in patients with mitochondrial myopathy (Figure 1). In addition, the regulatory data supporting their use *in vivo* are accumulating. In particular, pharmacokinetic parameters for NR in healthy volunteers have been recently reported, suggesting a dosing regimen of 1000 mg b.i.d., which is higher than previously used [51], and one clinical trial on myopathic patients is ongoing (Figure 1). It is still unclear, however, which the most effective strategy of stimulating mitochondrial biogenesis is. A second challenge is to develop suitable analytical methods to detect some of the compounds in blood, as they appear to have extremely short lifespan and to be degraded very quickly.

## Gene therapy approaches

### Gene replacement therapy

#### Rationale

The possibility of re-expressing the wild-type form of a missing or mutated gene is very attractive for all the genetic diseases, but this is currently a realistic goal only for those conditions affecting a single organ. Adeno-associated viral vectors (AAVs) are particularly attractive as delivery method because of their favourable safety profile and the availability of several tissue-specific serotypes. The main limitations concern the limited cloning capacity and the difficulty in achieving therapeutic expression levels in several tissues.

#### Results

AAVs have been used to deliver therapeutic genes in several mouse models of mitochondria disorders, including the models for ADOA [52], MNGIE [53], and EE [54].

For both MNGIE and EE, characterized by accumulation of toxic compounds [55], hepatotropic AAV8 vectors aimed at restoring the filter activity of the liver by re-expressing the missing gene. This very same goal was achieved in EE [56] and MNGIE [57] patients by using liver transplant. This allowed bypassing all the regulations needed to implement a clinical trial with AAVs. However, it should also be noted that pre-existing liver disease, as occasionally observed in MNGIE [57] and other diseases, may prevent the use of AAVs as cellular damage may interfere with viral entry into the cells. In addition, liver transplant has also been proposed as a suitable therapy for other mitochondrial diseases with hepatopathy, such as Alpers disease, provided that the clinical conditions are not too severe [58,59].

We recently showed that human *NDUFS4* was able to partially rescue the phenotype of *Ndufs4* KO mice only when simultaneously administered systemically and intracranially. This highlights the potential, but also the challenges of targeting multisystem disorders and as well as a complex organ as the brain [60]. In fact, the AAV9 serotype did not efficiently cross the blood–brain barrier, and mainly targeted glial cells when injected intracranially in newborns. Interestingly, new engineered serotypes showed great promises in their efficiency to cross the BBB [61]. Although this has been shown to be limited to rodents [62], future developments may isolate serotypes with similar tropism targeting human cells.

Several clinical trials (Figure 1) are ongoing using AAVs on LHON disease and one has been completed using allotropic expression of ND4, i.e. expression of mtDNA-encoded protein from the nucleus. Despite the absence of solid evidence regarding the import of mtDNA-encoded subunits into mitochondria and insertion into respiratory complexes, Guy et al. [63] reported amelioration of visual acuity in the injected eyes. Alternative explanation to this clinical effect could be the presence of a secondary mutation in mtDNA and a spontaneous recovery that is often observed especially in the m.11778G>A mutation [63].

A similar study was carried out using a single dose (5 × 10^9^ vg/0.05 ml) of rAAV2-ND4 on nine patients (NTC01267422 [64]). No adverse effects were recorded. In 6/9 patients, visual acuity improved and visual field was enlarged 9 months after treatment, while other parameters were unchanged.

An open-label Phase I/II clinical trial (NCT02064569) investigated the safety and preliminary efficacy of a rAAV2/2-*ND4* in four dose-escalation cohorts (9 × 10^9^, 3 × 10^10^, 9 × 10^10^, 1.8 × 10^11^ vector genomes/eye). Overall, the treatment proved to be safe as only mild to moderate adverse effects were reported by all the patients, such as increase in ocular pressure, ocular pain, vitritis. A clinically significant improvement in best-corrected visual acuity (i.e. the best vision achievable with the help of correction) was noted in the treated eyes of 6/14 subjects and a between-eye difference in visual acuity change from baseline was observed in a subset of patients with disease duration less than 2 years. A phase 3 study is currently ongoing on patients with vision loss for less than 6 months and between 7 months and 1 year (NCT03406104). Although the results have not yet been published, some have been made available to the public (https://www.businesswire.com/news/home/20180619006555/en/GenSight-Biologics-Key-Opinion-Leaders-Highlight-GS010) reporting a preservation of the retinal ganglion cell macular volume and nerve thickness and an improvement contrast sensitivity in treated eyes compared with the sham-treated ones. No difference was observed in high-contrast visual acuity. According to the sponsor, however, the intervention seemed to be more effective on young patients entering the treatment earlier, with vision loss less than 9 months. These results are promising but await confirmation and details to be made accessible to be properly evaluated.

#### Outlook

In spite of the difficulties related to the extremely high production costs and regulatory requirements, new stamina for the AAV-based gene therapy has been triggered by the positive results of a clinical trial on spinal muscular atrophy using AAV9, showing remarkable amelioration of the treated patients compared with the natural history of the disease [65]. It remains anyway challenging to address the safety concerns and comply with all the regulations as every single vector has to be treated as a new therapeutic agent.

### Molecular scissors to shift heteroplasmy

#### Rationale

The impossibility to manipulate mtDNA *in vivo* prevented for a long time the development of interventions aimed at modifying heteroplasmy levels. The introduction of TALENs and ZFN made a major step forward towards this end.

#### Results

Both mitochondrial targeted (mt) TALENs and mtZFNs were shown to be rather effective in shifting the heteroplasmy of several cellular models with mutations in mtDNA [66–68]. Interestingly, mitoZNFs were shown to prevent the germline transmission of mitochondrial mutations by inducing a heteroplasmy shift through the selective elimination of mutated mtDNA [69]. Very recently, two independent studies demonstrated the feasibility of this approach in vivo using a heteroplasmic mouse model carrying a point mutation in the tRNA for alanine. Both mtZFNs and mtTALENs were delivered by AAV vectors and proved to be able to significantly reduce heteroplasmy levels in heart, using mtZFNs, and skeletal muscle, respectively [70,71].

These strategies have been extensively reviewed elsewhere [72], but it is worth noting that the promises and potential of CRISPR/Cas9 system to manipulate nuclear genome do not apply to the manipulation of mtDNA due to the impossibility of importing RNAs (such as the guided RNAs which are integral components of the Cas9 nucleoprotein) into mitochondria. Importantly, other approaches based on import of therapeutic RNAs are equally very controversial [72].

#### Outlook

Although extremely promising, these approaches are still in their infancy and more work is warranted to fully assess their applicability to other models and to address safety issues. However, this is a clever approach that could, in principle, bypass some of the ethical concerns related to eliminate mtDNA mutations, such as mitochondrial replacement therapies (see below).

### Shaping mitochondria

#### Rationale

Opa1 is a GTPase of the inner mitochondrial membrane with key roles in regulating mitochondrial fusion as well as the structure of mitochondrial cristae. In 2013, Cogliati et al. [73] demonstrated that moderate overexpression of Opa1 increased the efficiency of the respiratory chain by regulating the physical and functional organization of the respiratory complexes into supercomplexes.

#### Results

We recently reported that moderate overexpression of *Opa1* was beneficial in models of mitochondrial encephalopathy and myopathy by stabilizing the defective complexes and supercomplexes [74]. Similarly, moderate Opa1 overexpression proved to be beneficial in several other conditions characterized by altered mitochondrial morphology, including denervation-induced muscle atrophy, liver damage, and ischaemic damage [75].

Additional strategies to increase mitochondrial fission/fusion have recently been proposed. For instance, Mfn2 agonists locking Mfn2 in the closed, inactive state thus promoting fusion, were identified and shown to effectively ameliorate mitochondrial abnormalities in cell lines carrying various mutations in MFN2, and to normalize axonal mitochondrial trafficking in sciatic nerves of MFN2 Thr^105 ^→ Met^105^ mice, a model of CMT2A [76]. Future work to assess their efficacy in models of OXPHOS deficiencies is warranted.

Another compound that was proposed to modify mitochondrial morphology is elamipretide (previously known as MTP-131). Elamipretide is a Szeto and Schiller tripeptide able to penetrate cells and to accumulate in mitochondria, where it is deemed to bind cardiolipin, a lipidic component of the inner mitochondrial membrane with an important role in regulating the RC activity and shaping mitochondrial cristae [77]. Although elamipretide has been shown to correct mitochondrial ultrastructure in cell models characterized by altered mitochondrial morphology, its mechanism is still poorly understood and no evidence that it may be effective in primary mitochondrial dysfunction has been provided. However, several clinical trials with elamipretide are currently ongoing for primary mitochondrial disease, primary mitochondrial myopathy, and LHON. Only for one of them (NCT02367014), aimed at providing evidence for safety and initial efficacy assessment, the results have been recently published [78]. Elamipretide showed substantially favourable safety profile and improved the 6-minute-walking test in the treated group compared with the placebo group.

#### Outlook

The correction of mitochondrial ultrastructure and the intervention to increase mitochondrial fusion are very promising approaches, but several issues need to be address to move towards the clinics*.* First, it is currently unknown whether prolonged activation of Opa1 can have toxic effects. Second, only a few compounds have been shown to modify fission/fusion processes and/or to shape mitochondrial cristae through Opa1-dependent or -independent processes. Future work will need to find new approaches to tune the activity of Opa1 or other proteins of the fission/fusion machinery.

## Pre-implantation genetic diagnosis and mitochondrial replacement therapy

### Rationale

Pre-implantation genetic diagnosis is offered in specialized centres, but it has the limit of potential heteroplasmy levels drifting in the embryo over time leading to disease. Since mtDNA mutations are maternally inherited, the transmission of pathogenic mutations can be prevented by reproductive techniques aimed at replacing the mitochondria in oocytes of carrier women.

### Results

Two techniques have been developed to avoid transmission of mutations in mtDNA [79,80]. The first has been developed in the U.K., where the treatment is now licensed for use in humans, and is based on the pronuclear transfer from an affected donor into an enucleated healthy embryo shortly after completion of meiosis [81]. Importantly, the carryover-mutated mtDNA was less than 2% in the majority of the embryos, reducing the probability of a later proliferation of mutant mtDNA, as observed when mutant DNA carryover was above 4%. The alternative approach, developed in the U.S.A., is based on the maternal spindle transfer, in which the nucleus of the affected mother's oocyte is transferred to an enucleated donor's oocyte *before* fertilization with the father's sperm [82]. In this case, the mutated mtDNA carryover was ∼1%, although some stem cell lines generated in the present study progressively lost the donor mtDNA reversing to the maternal one, possibly related to specific haplotypes conferring a replicative advantage, as suggested by the presence of a specific polymorphism in the D-loop of the preferred haplotype. The first baby born by using the spindle transfer technique was reported last year in a highly controversial article [83]. Ethical and technical issues were raised in the editorial comment and further articles. Consent form and informative material were not tailored to the procedure and a two-step procedure was performed under two different legislations (US and Mexico) raising major ethical concerns. From technical point of view, details regarding the use of electrofusion and explanation of high rate degeneration of oocytes were missing. Finally, although positive, the outcomes of the heteroplasmy levels were more than 2% of carryover and therefore not optimal to assure an absent or very low risk to develop disease [84].

### Outlook

Despite the technical and ethical concerns that have challenged and will continue to challenge the mitochondrial replacement, this ‘world-first birth' represented a pivotal step for translating the therapy in clinical trial. In 2015, UK regulatory bodies have approved the use of mitochondrial replacement therapy for use in selected patients by embryologists with high level of expertise with a planned long-term follow-up of the children born [85].

## Towards the development of clinical trials

Preclinical studies are opening the way to have effective and safe translatable cure for the patients. Therefore, there is an urgency to identify clinical end-points, accurate biomarkers, and appropriate outcome measures for designing a clinical trial. The first major effort of the scientific community has been in the study of disease natural history defined as ‘natural course of a disease from the time immediately prior to its inception, progressing through its pre-symptomatic phase and different clinical stages to the point where the disease has ended without external intervention' [86]*.* In a systematic literature review, Rahman et al. identified 35 natural history studies encompassing 28 mitochondrial disease entities. Data from those studies have helped to redefine diagnostic criteria for classical clinical syndromes and to establish a clinical baseline for comparison in single-arm clinical trials of novel therapies [87]. However, the majority of the natural history studies are currently based on retrospective analysis of previously diagnosed cohort of patients with limits in data quality, and this has recently emerged as a primary need in the field [88]. In addition, clinical rating scales dedicated to mitochondrial patients such International Pediatric Mitochondrial Disease and Newcastle Mitochondrial Disease Adult Scales and motor functional and quality-of-life scales adopted from previous trials in neuromuscular disorders have been validated for clinical trial readiness in mitochondrial disorders [88].

## Conclusions

The development of new therapies for mitochondrial diseases is moving fast at the preclinical level, but the clinical translation is lagging behind. The only treatment approved by the European Medicines Agency is currently based on the use of idebenone for LHON. Interestingly, relatively large clinical trials have supported its efficacy in LHON patients, underscoring the necessity of rigorously designed trials to determine the efficacy of new drugs, a goal often difficult to achieve given the rarity of most of the syndromes. However, an increasing number of clinical trials are being implemented and we expect major steps forward in the coming years.

## Abbreviations

AAVs: Adeno-associated viral vectors
ADOA: dominant optic atrophy
AICAR: 5-Aminoimidazole-4-carboxamide ribonucleotide
AMPK: AMP-dependent kinase
ATP: adenosine triphosphate
BBB: blood–brain barrier
Cas9: CRISPR associated protein 9
CMT2A: Charcot-Marie-Tooth type 2A
CoQ: coenzyme Q
COX: cytochrome c oxidase
CRISPR: Clustered Regularly Interspaced Short Palindromic Repeats
DGUOK: deoxyguanosine kinase
dNTPs: deoxyribonucleoside triphosphates
EE: ethylmalonic encephalopathy
HIFs: hypoxia-induced transcription factors
iPSCs: induced pluripotent stem cells
KD: ketogenic diet
LHON: Leber's hereditary optic neuropathy
LS: Leigh syndrome
Mfn2: mitofusin 2
MILS: maternally inherited Leigh syndrome
MNGIE: mitochondrial neuro-gastro-intestinal encephalo-myopathy
mtDNA: mitochondrial DNA
mtISR: mitochondrial integrated stress response
mTOR: mechanistic target of rapamycin
NAC: *N*-acetyl cysteine
NAD^+^: nicotinammide adenina dinucleotide
NR: nicotinamide riboside
Opa1: Optic atrophy 1
OXPHOS: oxidative phosphorylation
PEO: progressive external ophthalmoplegia
RRM2B: p53-inducible ribonucleotide reductase small subunit
rRNA: ribosomal RNA
TALEN: Transcription activator-like effector nucleases
*Tk2*: thymidine kinase
tRNA: transfer RNA
*Tymp*: thymidine phosphorylase
*Upp1*: uridine phosphorylase 1
VHL: von Hippel–Lindau
ZFN: zinc fingers nucleases
